# Galectin-10 and Charcot-Leyden Crystals in Chronic Rhinosinusitis with Nasal Polyps: Pathogenetic Insights and Clinical Implications

**DOI:** 10.3390/diagnostics16172737

**Published:** 2026-08-26

**Authors:** Bartłomiej Kamiński, Dominika Ochab, Janusz Kopczyński, Piotr Łacwik, Cezary Pałczyński

**Affiliations:** 1 Department of Otolaryngology, Maria Skłodowska-Curie District Hospital, 26-110 Skarżysko-Kamienna, Poland; 2 Collegium Medicum, Jan Kochanowski University, 25-369 Kielce, Poland; 3 Clinical Division of Lung Diseases and Allergology, Holy Cross Centre for Lung Diseases, 26-060 Czerwona Góra, Poland; 4 Department of Oncological Pathology, Holy Cross Cancer Centre, 25-734 Kielce, Poland

**Keywords:** chronic rhinosinusitis with nasal polyps, galectin-10, Charcot-Leyden crystals, eosinophils, type 2 inflammation, eosinophil extracellular trap cell death (EETosis), biomarkers, biologic therapy

## Abstract

Chronic rhinosinusitis with nasal polyps (CRSwNP) is a heterogeneous inflammatory disease in which type 2 inflammation predominates in most patients in Western populations. Galectin-10 (Gal-10) and Charcot-Leyden crystals (CLCs), linked to eosinophil activation, cytolysis and eosinophil extracellular trap cell death (EETosis), have emerged as potential biomarkers and mediators of eosinophilic inflammation. This narrative review summarises current evidence on their biological role and clinical relevance in CRSwNP. The literature was identified through searches of PubMed/MEDLINE, Web of Science and Google Scholar, with emphasis on mechanistic studies, clinical investigations and recent evidence concerning type 2 inflammatory airway diseases. Available evidence suggests that Gal-10 and CLCs are associated with eosinophil activation and may contribute to persistent type 2 inflammation through epithelial injury, increased mucus viscosity, impaired mucociliary clearance, tissue remodelling and inflammatory amplification. Gal-10/CLCs may have value as investigational biomarkers of disease activity, postoperative recurrence and response to biologic therapy. Current biologics may indirectly modulate the Gal-10/CLC pathway by reducing eosinophil survival, recruitment or activation, although direct evidence remains limited. Gal-10 and CLCs are promising investigational biomarkers and mechanistically plausible therapeutic targets in CRSwNP. However, clinical application is limited by the lack of standardised analytical methods, validated cut-off values and prospective studies demonstrating incremental value beyond established biomarkers. Further research is required to clarify their role in stratification, prognosis and monitoring of biologic therapy.

## 1. Introduction

According to EPOS 2020, chronic rhinosinusitis with nasal polyps (CRSwNP) is a heterogeneous disease in which type 2 inflammation represents one of the key pathophysiological mechanisms. In Western populations, the type 2 endotype is present in a substantial proportion of patients with CRSwNP and, according to some data, may occur in up to approximately 80% of patients with nasal polyps [1].

Type 2 inflammation involves a complex immune response mediated by Th2 lymphocytes, group 2 innate lymphoid cells (ILC2s), eosinophils, mast cells and basophils. Type 2 cytokines, particularly interleukin-4 (IL-4), interleukin-5 (IL-5) and interleukin-13 (IL-13), play a central role. IL-4 and IL-13 contribute to immunoglobulin class switching towards IgE production, epithelial activation, chemokine induction and tissue remodelling, whereas IL-5 regulates eosinophil differentiation, survival, activation and recruitment to airway tissues [1,2].

Persistent type 2 inflammation leads to epithelial barrier dysfunction, inflammatory-cell infiltration and remodelling of the extracellular matrix. In CRSwNP, impaired local fibrinolysis is also important and is related, among other mechanisms, to reduced activity of tissue plasminogen activator (t-PA). This results in fibrin deposition, fluid retention, stromal oedema and formation of a characteristic tissue matrix that favours the development of nasal polyps [2,3].

In clinical practice, EPOS 2020 recommends identifying type 2 inflammation on the basis of available clinical features and biomarkers. The most commonly used indicators include tissue eosinophilia of ≥10 eosinophils per high-power field, peripheral blood eosinophilia of ≥250 cells/μL and total IgE concentration of ≥100 IU/mL [1].

CRSwNP with a type 2 endotype frequently coexists with other type 2 inflammatory diseases, particularly asthma, non-steroidal anti-inflammatory drug-exacerbated respiratory disease (N-ERD) and eosinophilic otitis media [3,4,5]. Identification of the type 2 endotype is important not only for understanding disease pathophysiology, but also for selecting patients for biologic therapy.

Eligibility for biologic therapy in CRSwNP is multidimensional and should not be reduced to the presence of type 2 inflammation alone. It includes assessment of disease severity, persistence of symptoms despite optimal medical treatment, history of previous endoscopic sinus surgery, need for systemic corticosteroids, olfactory dysfunction, impact on quality of life, comorbid asthma and evidence of type 2 inflammation [1,6].

Biologic therapies used in CRSwNP target different elements of type 2 inflammation. Dupilumab blocks the α subunit of the IL-4 receptor, thereby inhibiting IL-4- and IL-13-mediated signalling. Mepolizumab neutralises free IL-5, whereas benralizumab binds the IL-5 receptor α subunit and leads to profound eosinophil depletion. Omalizumab binds free IgE and limits IgE-dependent inflammatory pathways. More recently, upstream epithelial alarmin signalling has become an important therapeutic target, particularly through inhibition of thymic stromal lymphopoietin (TSLP) with tezepelumab. Differences in the molecular targets of these agents may be relevant to their potential effects on eosinophil activation, Gal-10 release and Charcot-Leyden crystal formation [6,7,8,9,10].

The aim of this narrative review is to summarise current evidence on the biological role of Gal-10 and Charcot-Leyden crystals in eosinophilic type 2 inflammation, with particular emphasis on CRSwNP. The review discusses the structure and biological properties of Gal-10, mechanisms of Gal-10 release and CLC formation, the potential contribution of CLCs to inflammatory amplification, and the emerging relevance of Gal-10/CLCs as investigational biomarkers of disease activity, recurrence risk and response to biologic therapy. In addition, current analytical and clinical limitations of Gal-10/CLC assessment are discussed to clarify the extent to which these markers may be considered research tools rather than established clinical biomarkers.

## 2. Materials and Methods

This article is a narrative review. The literature was identified through searches of PubMed/MEDLINE, Web of Science and Google Scholar. The following search terms were used alone and in combination: “galectin-10”, “Charcot-Leyden crystals”, “eosinophil extracellular traps”, “EETosis”, “chronic rhinosinusitis with nasal polyps”, “CRSwNP”, “type 2 inflammation”, “eosinophilic inflammation”, “biomarkers”, “biologic therapy”, “dupilumab”, “mepolizumab”, “benralizumab”, “omalizumab” and “tezepelumab”.

Articles published in English were preferentially included, with emphasis on mechanistic studies, clinical studies in CRSwNP and eosinophilic airway diseases, biomarker studies and relevant reviews. Additional publications were identified from the reference lists of selected articles. Because this was not a systematic review, no formal risk-of-bias assessment or meta-analysis was performed. The available evidence was interpreted narratively, with particular attention to biological plausibility, clinical relevance and the limitations of current knowledge.

## 3. Biomarkers of Type 2 Inflammation in CRSwNP

In addition to classical indicators of type 2 inflammation, such as tissue eosinophilia, peripheral blood eosinophilia and total IgE concentration, increasing attention is being paid to biomarkers reflecting effector-cell activation and local inflammatory activity. In the future, these markers may complement assessment of disease endotype, recurrence risk after surgery and response to biologic therapy. However, it should be emphasised that most of these markers, including Gal-10 and Charcot-Leyden crystals, remain investigational and require further validation before routine clinical use [1,6,11,12,13,14,15,16].

Biomarkers related to eosinophil activity include eosinophil cationic protein (ECP), eosinophil-derived neurotoxin (EDN), major basic protein (MBP), eosinophil peroxidase (EPX), galectin-10 (Gal-10) and Charcot-Leyden crystals (CLCs). In contrast to eosinophil counts alone, these markers may provide information on effector-cell activation, degranulation, cell death and the presence of extracellular inflammatory structures in airway tissue or secretions [11,12,14,15,16].

Galectin-10 and CLCs are currently being investigated as potential markers of eosinophilic inflammatory activity and possible mediators of persistent chronic type 2 inflammation. Their assessment may provide information on eosinophil activation, EETosis and the local inflammatory microenvironment; however, the clinical significance of Gal-10/CLCs requires assay standardisation, validation of cut-off values and comparison with established biomarkers [13,14,15,16].

Another non-invasive marker of type 2 inflammation is the fraction of exhaled nitric oxide (FeNO), which is traditionally used in the diagnosis and monitoring of asthma. In patients with CRSwNP, particularly those with comorbid asthma, FeNO may reflect type 2 inflammatory activity within the united airways. This marker may support patient phenotyping, although its value in the isolated assessment of sinonasal inflammation remains limited and should be interpreted in the context of clinical findings and other parameters [6,17].

Periostin, an extracellular matrix protein induced by IL-4 and IL-13, is another potential biomarker of type 2 inflammatory activity and tissue remodelling. Increased periostin concentrations in serum or nasal secretions may reflect activation of the IL-4/IL-13 axis and enhanced mucosal remodelling. However, as with other biomarkers, further studies are required to determine its predictive value and clinical usefulness [18,19].

In recent years, epithelial alarmins such as TSLP, IL-33 and IL-25 have also attracted increasing interest. These mediators are released by damaged or activated epithelium and represent early signals initiating the inflammatory response in the airways. In type 2 inflammation, alarmins link epithelial barrier dysfunction with activation of ILC2s, Th2 lymphocytes, mast cells, basophils and eosinophils. The relevance of TSLP has increased further with the development of anti-TSLP therapy. Tezepelumab has demonstrated efficacy in severe uncontrolled CRSwNP, including reductions in nasal polyp size, nasal congestion, sinonasal symptoms, the need for surgery and systemic corticosteroid use. However, direct evidence that tezepelumab modulates Gal-10 release, EETosis or CLC formation in CRSwNP remains limited [20].

Recent studies also indicate the importance of metabolic changes and remodelling within the nasal polyp microenvironment. One of the analysed pathways is the hypoxia-inducible factor 1-alpha (HIF-1α) axis, which may contribute to metabolic reprogramming of stromal cells, oedema, fibrosis and formation of the characteristic extracellular matrix of nasal polyps. These data are consistent with the concept of more precise, multiparametric profiling of CRSwNP [21].

## 4. Structure and Biological Properties of Gal-10

Galectin-10 belongs to the galectin family and contains a carbohydrate recognition domain; however, compared with many other galectins, it displays atypical glycan-binding properties. Gal-10 is one of the major cytoplasmic proteins of human eosinophils and accounts for approximately 7–10% of the total cytoplasmic protein content of these cells. It is found mainly in the cytoplasm but may also be present in nuclear compartments and membrane-associated structures [12,15,22,23].

Gal-10 is strongly expressed primarily in eosinophils and basophils. Reports of Gal-10 detection in other inflammatory cells should be interpreted with caution, as they may reflect extracellular protein uptake, cell–cell interactions or methodological differences rather than constitutive endogenous Gal-10 expression in these cells [15,22,23].

The precise intracellular function of Gal-10 remains incompletely understood. This protein may be involved in processes related to eosinophil maturation and activation, organisation of cellular compartments, cell death and inflammatory-cell interactions. One of its most important biological features is its ability to crystallise under conditions of high local concentration, particularly after eosinophil cytolysis or EETosis [15,24,25].

## 5. EETosis and Gal-10 Release

Understanding the role of Gal-10 and EETosis, that is, eosinophil cell death associated with the formation of extracellular eosinophil traps, is important for explaining the chronic and recurrent nature of eosinophilic inflammation in CRSwNP. Traditionally, tissue injury in eosinophilic diseases was attributed mainly to classical eosinophil degranulation and the release of toxic granule proteins. Currently, increasing attention is also being given to eosinophil cytolysis, extracellular trap formation and Gal-10 crystallisation [22,26,27,28].

EETosis and Gal-10 crystallisation are considered mechanisms that may contribute to the persistence of chronic eosinophilic inflammation. However, they should not be regarded as a universal and fully established sequence of events occurring in all patients with CRSwNP. Eosinophil activation may lead to different cellular responses, including classical degranulation, cytolysis, EETosis, vital extracellular trap formation, apoptosis or secondary necrosis. Consequently, Gal-10 may be released through different pathways, and not all extracellular Gal-10 necessarily undergoes crystallisation [22,23,26,28].

During EETosis, the nuclear envelope breaks down, chromatin decondenses, and nuclear material together with effector proteins is released into the extracellular space. The resulting extracellular eosinophil traps consist of a DNA scaffold associated with eosinophil granule proteins such as ECP, MBP and EDN, as well as Gal-10. In CRSwNP, these structures may be present in mucus and in the nasal polyp stroma, particularly in areas of epithelial barrier damage and pronounced eosinophilic inflammation [26,27].

## 6. Formation of Charcot-Leyden Crystals

CLC formation is thought to be associated with local accumulation of Gal-10 in the extracellular space after eosinophil cytolysis, EETosis or other processes related to cell activation and damage. Under conditions of high local Gal-10 concentration, ordered self-association of Gal-10 molecules may occur, resulting in the formation of characteristic colourless bipyramidal structures [12,14,24,25,28].

The exact concentration threshold required for spontaneous Gal-10 crystallisation in human airway secretions has not been clearly established and may depend on local protein concentration, pH, ionic conditions, mucus composition, the presence of extracellular DNA and other inflammatory proteins [14,24,25].

Charcot-Leyden crystals were first described in the nineteenth century in tissue and secretion samples associated with eosinophil-rich diseases [29,30]. For many years, they were regarded mainly as microscopic markers of eosinophilic inflammation. More recent studies suggest, however, that CLCs may not only reflect the presence of eosinophils but may also actively participate in sustaining inflammation [14,24,25,28].

## 7. CLCs, the NLRP3 Inflammasome and Amplification of Inflammation

CLCs may act as crystalline danger-associated molecular patterns. After phagocytosis by macrophages or dendritic cells, insoluble crystals may cause lysosomal destabilisation and activation of the NLRP3 inflammasome, followed by release of IL-1β and IL-18. This mechanism may link eosinophilic type 2 inflammation with additional inflammatory amplification pathways [14,25,31].

It may also contribute to the development of a self-perpetuating inflammatory loop in which eosinophil recruitment and activation lead to further Gal-10 release and CLC formation. However, the relevance of this mechanism in human CRSwNP requires further confirmation. Of particular interest are experimental data suggesting that antibody-mediated dissolution of CLCs may reduce established inflammation. At present, however, anti-CLC strategies remain experimental and have no confirmed clinical application in CRSwNP [14,25,31].

## 8. Relevance of Gal-10/CLCs in CRSwNP and Eosinophilic Diseases

The accumulation of extracellular Gal-10, EETs and CLCs may contribute to selected clinical features of CRSwNP. These structures may enhance epithelial barrier damage, increase mucus viscosity, impair mucociliary clearance and promote oedema and tissue remodelling. In the context of nasal polyps, the influence of eosinophilic inflammation on fibrin metabolism, fibrin deposition and stromal fluid retention may also be important [2,13,22,32].

**Figure 1:**
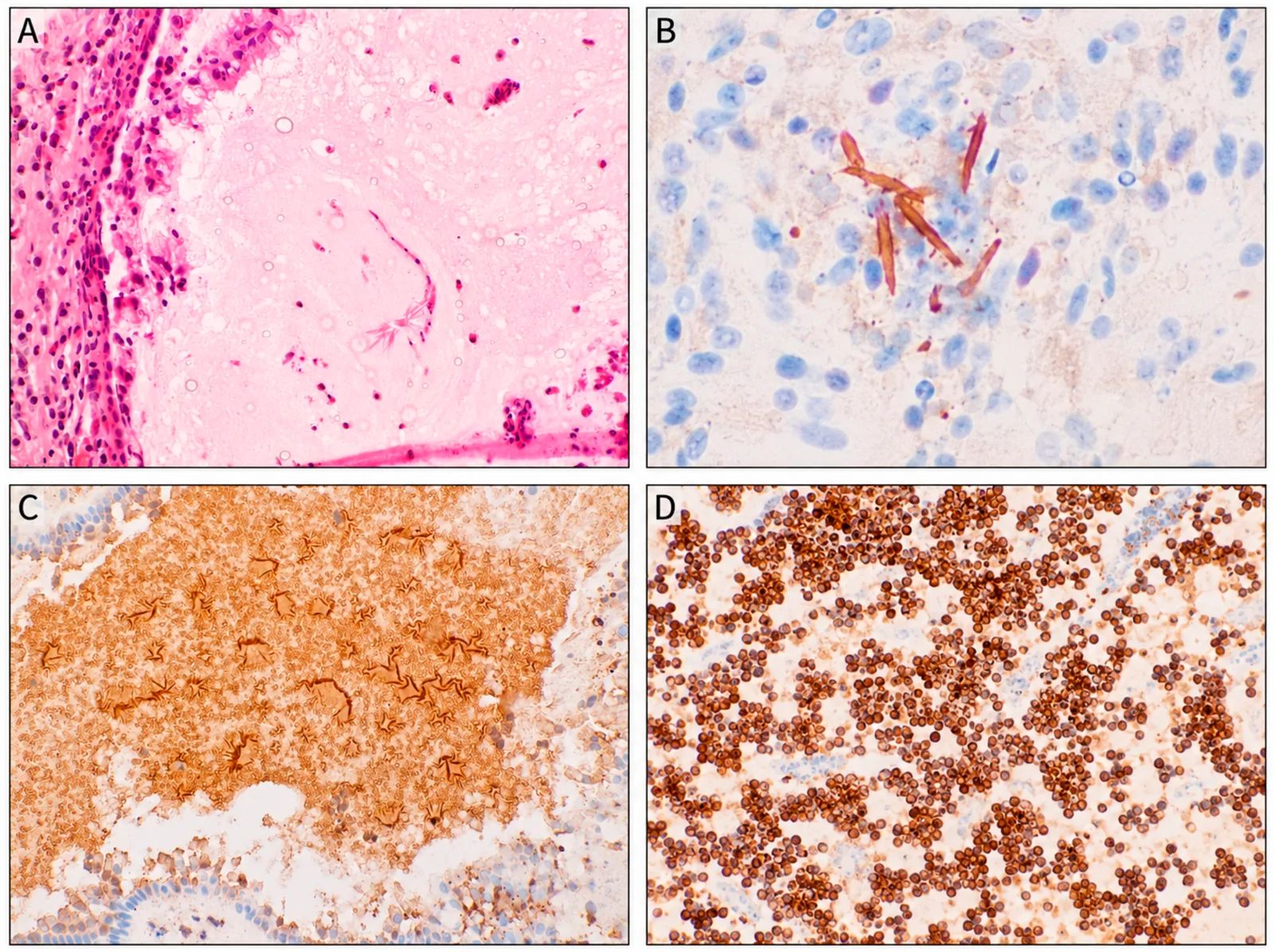
Histopathological and immunohistochemical images illustrating Charcot-Leyden crystals (CLCs) and galectin-10 (Gal-10) in nasal secretions from a patient with CRSwNP. (**A**) Hematoxylin and eosin staining showing characteristic CLCs in nasal mucus/secretions. (**B**) Higher-magnification immunohistochemical image demonstrating Gal-10-positive crystal structures. (**C**) Lower-magnification view showing abundant extracellular Gal-10-positive material with multiple CLCs. (**D**) Immunohistochemical staining showing both intracellular and extracellular Gal-10 expression in inflammatory cells and secretions. Brown staining indicates Gal-10 positivity.

The effect of Gal-10 and CLCs on mucus properties may be both direct and indirect. In eosinophilic inflammation, extracellular DNA, eosinophil granule proteins, Gal-10, CLCs and mucins such as MUC5AC and MUC5B may form a dense inflammatory matrix that increases mucus viscosity. This may impair mucociliary transport and contribute to the persistence of chronic inflammation. Experimental data also suggest that Gal-10 may influence the expression of genes related to mucin production; however, direct biochemical interactions between CLCs and individual mucin polymers in human CRSwNP require further investigation [14,33,34]. Representative histopathological and immunohistochemical images illustrating CLCs and Gal-10-positive material in nasal secretions are shown in Figure 1.

The presence of EETs and CLCs within the nasal and sinus mucosa, potentially including the olfactory epithelium region, may be one of the mechanisms contributing to olfactory dysfunction in patients with CRSwNP. It should be emphasised, however, that smell loss in CRSwNP is multifactorial and may result from mechanical nasal obstruction, inflammation of the olfactory epithelium, oedema, tissue remodelling and local activity of inflammatory mediators [22,32].

Chen et al. evaluated the significance of CLC structures in predicting nasal polyp recurrence after ESS [22]. The authors showed that the presence and number of CLCs in tissue may have prognostic value; for a defined cut-off value, high sensitivity and specificity for predicting recurrence were reported. These findings are important because they provide quantitative data on the potential predictive value of CLCs. However, a single study is insufficient to establish CLCs as validated clinical biomarkers, and the reported cut-off values require external validation in independent cohorts [22].

The relevance of Gal-10 extends beyond CRSwNP. In eosinophilic otitis media, numerous eosinophils and positive immunohistochemical staining for Gal-10 have been reported in middle-ear effusion samples. These observations support the concept of a shared eosinophilic type 2 inflammatory mechanism involving the upper and lower airways and the middle ear [4,5].

## 9. Gal-10/CLCs and Biologic Therapy

Biologic therapy may affect the Gal-10/CLC pathway indirectly by influencing eosinophil number, survival, recruitment or activation. However, it should be emphasised that direct data on Gal-10/CLCs as endpoints in CRSwNP remain limited, and most conclusions are mechanistic or hypothetical [6,7,8,9,10,23,35,36,37,38].

Mepolizumab neutralises free IL-5, a key cytokine regulating eosinophil survival, activation and recruitment. Because Gal-10 is an abundant cytoplasmic protein of eosinophils, reducing the number of these cells may secondarily limit the total pool of cells capable of releasing Gal-10. Consequently, anti-IL-5 treatment may potentially decrease Gal-10 availability in the inflammatory microenvironment and limit the formation of new CLCs. However, direct data on the effect of mepolizumab on Gal-10/CLCs in nasal polyp tissue remain limited [7,35,36].

Benralizumab acts by binding the IL-5 receptor α subunit and inducing antibody-dependent cell-mediated cytotoxicity, leading to profound eosinophil depletion. Therefore, its potential effect on Gal-10/CLCs may result mainly from reduction in the number of cells capable of releasing Gal-10. In contrast to mepolizumab, which neutralises free IL-5, benralizumab acts at the level of the target cell through IL-5Rα, which justifies its separate discussion in the context of the Gal-10/CLC pathway [9].

Dupilumab blocks the α subunit of the IL-4 receptor, thereby inhibiting IL-4- and IL-13-mediated signalling. These cytokines are not the main regulators of eosinophil differentiation in the bone marrow, but they play an important role in epithelial activation, eotaxin production, adhesion-molecule expression and recruitment of eosinophils to tissues. Therefore, blockade of the IL-4/IL-13 pathway may limit eosinophil influx and activation in the nasal and sinus mucosa. Transcriptomic studies suggest that dupilumab treatment may be associated with reduced expression of the CLC/Gal-10 gene in nasal tissue. However, it cannot currently be concluded that modulation of Gal-10/CLCs is the principal mechanism of dupilumab’s clinical efficacy [8,37,38].

Omalizumab binds free IgE and limits IgE-dependent pathways, including activation of mast cells and basophils. Its effect on eosinophils, Gal-10 and CLCs is probably more indirect than that of agents targeting the IL-5/IL-5R or IL-4/IL-13 pathways. Potential reduction in type 2 inflammatory activity may secondarily limit eosinophil activation; however, direct data on the effect of omalizumab on Gal-10/CLCs in CRSwNP are limited [10].

Tezepelumab, a monoclonal antibody targeting TSLP, represents an upstream therapeutic approach in type 2 inflammatory airway disease. By inhibiting epithelial alarmin signalling, it may indirectly reduce eosinophil recruitment, activation, EETosis and subsequent Gal-10 release or CLC formation. However, tezepelumab does not directly target Gal-10 or preformed CLCs, and direct evidence regarding its effect on Gal-10/CLC-related pathways in CRSwNP remains limited. Thus, Gal-10/CLCs may remain relevant as downstream markers of residual eosinophilic tissue activity in the era of upstream biologic therapy. The study by Qi et al. indicates that measurement of CLC gene mRNA in nasal brushing samples may represent a potential minimally invasive approach to molecular profiling in CRSwNP. Increased CLC expression was associated with the presence of CRSwNP and a higher risk of polyp recurrence after ESS. In turn, data from Dorismond et al. suggest that dupilumab treatment may influence CLC/Gal-10 expression in nasal tissue. Taken together, these studies indicate the potential relevance of Gal-10/CLCs in assessing eosinophilic inflammatory activity; however, they do not yet confirm their role as standalone validated prognostic biomarkers or markers for monitoring biologic therapy [23,37,38].

The potential effects of biologic therapies on type 2 inflammation and the Gal-10/CLC pathway are summarised in Table 1.

## 10. Current Limitations and Future Perspectives of Gal-10/CLC Assessment

At present, Gal-10/CLC assessment should be regarded as a promising research tool rather than an established clinical biomarker. Available studies suggest associations between Gal-10/CLCs and eosinophilic inflammation, disease severity, recurrence risk after endoscopic sinus surgery (ESS) or molecular changes during biologic therapy. However, several requirements necessary for a biomarker to be implemented in routine clinical practice remain unmet [13,14,15,16,22,23].

The most important limitations include lack of standardisation of sampling and sample-processing methods, absence of clearly defined and externally validated cut-off values, limited data on biological variability, insufficient assessment of inter-laboratory reproducibility and incomplete data on sensitivity, specificity, positive predictive value and negative predictive value. Moreover, it has not yet been clearly demonstrated whether Gal-10/CLC measurement provides incremental value beyond established parameters such as tissue eosinophilia, peripheral blood eosinophils, total IgE, FeNO, periostin, endoscopic findings, olfactory testing and clinical questionnaires [13,14,15,16,17,22,23].

An important analytical challenge is that Gal-10 may be present both as a soluble protein and in the form of insoluble Charcot-Leyden crystals. Standard immunoassays, including enzyme-linked immunosorbent assay (ELISA)-based methods, may underestimate total Gal-10 levels if crystalline material is not adequately dissolved or lysed before analysis. Results may depend on specimen type, sampling site, storage conditions, extraction buffers and lysis or solubilisation protocols. Therefore, before possible clinical implementation, validated and reproducible methods for Gal-10/CLC measurement are required across different types of material, including nasal polyp tissue, nasal lavage fluid, mucus, airway secretions and nasal brushing samples [11,14,15,16,22,23].

## 11. Conclusions

Gal-10 and Charcot-Leyden crystals are emerging as promising research biomarkers and potential mediators of eosinophilic type 2 inflammation in CRSwNP. Available evidence suggests that they may reflect eosinophil activation, EETosis and persistence of the inflammatory microenvironment of the mucosa. However, their clinical utility remains insufficiently validated [13,14,15,16,22,23].

Future studies should focus on standardising sampling and sample-processing methods, defining externally validated cut-off values, assessing biological variability and determining whether Gal-10/CLC measurement provides incremental value beyond established clinical and laboratory parameters, such as tissue eosinophilia, peripheral blood eosinophils, total IgE, FeNO, periostin, endoscopic findings, olfactory assessment and clinical questionnaire scores [13,14,15,16,17,22,23].

Until such data are available, Gal-10/CLCs should be regarded as research tools rather than routine clinical biomarkers. At the same time, further investigation of the Gal-10/CLC pathway may improve understanding of the mechanisms underlying eosinophilic inflammation, CRSwNP recurrence and response to biologic therapy [14,22,23,24,25,37,38].

## Figures and Tables

**Table 1:** Potential effects of biologic therapies on type 2 inflammation and the Gal-10/CLC pathway.

Feature/Biomarker	Mepolizumab	Benralizumab	Dupilumab	Omalizumab	Tezepelumab
Target	Free IL-5	IL-5 receptor α subunit	IL-4Rα subunit, blocking IL-4 and IL-13 signalling	Free IgE	TSLP
Main mechanism	Reduces IL-5-mediated eosinophil survival and activation	Induces eosinophil depletion through antibody-dependent cell-mediated cytotoxicity	Reduces IL-4/IL-13-mediated epithelial activation, eotaxin production and eosinophil tissue recruitment	Reduces IgE-mediated mast-cell and basophil activation and downstream type 2 inflammation	Blocks upstream epithelial alarmin signalling and modulates downstream type 2 inflammatory pathways
Blood eosinophil count	Marked decrease	Profound and sustained decrease	Possible transient increase, followed by stabilisation or decrease	Usually no direct eosinophil-depleting effect; variable indirect effects	Possible indirect reduction or attenuation depending on baseline inflammatory profile
Tissue eosinophil count	Decrease related to IL-5 inhibition	Marked decrease related to IL-5Rα targeting and eosinophil depletion	Decrease related to reduced eosinophil recruitment into tissues	Possible indirect reduction through attenuation of IgE-mediated inflammation	Potential indirect reduction through decreased epithelial alarmin-driven type 2 signalling
Potential effect on Gal-10 release	May reduce the total eosinophil pool capable of releasing Gal-10	May strongly reduce the eosinophil pool capable of releasing Gal-10 because of eosinophil depletion	May reduce local Gal-10 release by limiting eosinophil recruitment and activation in mucosal tissue	May indirectly reduce Gal-10 release if IgE-driven type 2 inflammation and eosinophil activation are attenuated	Hypothetical indirect reduction through decreased eosinophil recruitment and activation
Potential effect on EETosis	Indirect reduction through decreased eosinophil survival and activation	Indirect reduction through eosinophil depletion	Reduction through modification of the IL-4/IL-13-driven inflammatory microenvironment	Indirect and less clearly established	Hypothetical indirect reduction through attenuation of upstream epithelial alarmin signalling
Potential effect on CLC formation	Potential limitation of new CLC formation due to reduced eosinophil availability	Potentially strong limitation of new CLC formation due to eosinophil depletion	Potential reduction through decreased tissue eosinophil activation and reduced CLC/Gal-10 gene expression	Uncertain; possible indirect effect through reduced IgE-mediated type 2 inflammation	Uncertain; possible indirect limitation of new CLC formation if eosinophilic tissue inflammation is reduced
Main limitation of evidence	Limited direct data on Gal-10/CLC outcomes in CRSwNP	Very limited direct data on Gal-10/CLC outcomes in CRSwNP	Some transcriptomic evidence suggests reduced CLC/Gal-10 gene expression, but causality remains uncertain	Direct data on Gal-10/CLC modulation are lacking	Clinical efficacy in CRSwNP has been demonstrated, but direct data on Gal-10/CLC modulation are lacking

Note: The proposed effects on Gal-10, EETosis and CLCs represent mechanistic hypotheses based on the known targets of biologic therapies and available data on eosinophilic inflammation. Direct comparative data on the effects of individual biologics on the Gal-10/CLC pathway in CRSwNP remain limited.

## Data Availability

No new data were created or analysed in this study. Data sharing is not applicable to this article.

## Abbreviations

The following abbreviations are used in this manuscript:

CLC	Charcot-Leyden crystal
CRSwNP	Chronic rhinosinusitis with nasal polyps
ECP	Eosinophil cationic protein
EDN	Eosinophil-derived neurotoxin
EETosis	Eosinophil extracellular trap cell death
EPOS	European Position Paper on Rhinosinusitis and Nasal Polyps
EPX	Eosinophil peroxidase
ESS	Endoscopic sinus surgery
FeNO	Fractional exhaled nitric oxide
Gal-10	Galectin-10
IgE	Immunoglobulin E
IL	Interleukin
ILC2	Group 2 innate lymphoid cell
MBP	Major basic protein
N-ERD	NSAID-exacerbated respiratory disease
TSLP	Thymic stromal lymphopoietin
